# Cellular and Humoral Cross-Immunity against Two H3N2v Influenza Strains in Presumably Unexposed Healthy and HIV-Infected Subjects

**DOI:** 10.1371/journal.pone.0105651

**Published:** 2014-08-27

**Authors:** Chiara Agrati, Concetta Castilletti, Eleonora Cimini, Daniele Lapa, Serena Quartu, Claudia Caglioti, Simone Lanini, Giovanni Cattoli, Federico Martini, Giuseppe Ippolito, Maria R. Capobianchi

**Affiliations:** 1 Laboratory of Virology, National Institute for Infectious Diseases “Lazzaro Spallanzani” I.R.C.C.S., Rome, Italy; 2 Laboratory of Cellular Immunology, National Institute for Infectious Diseases “Lazzaro Spallanzani” I.R.C.C.S., Rome, Italy; 3 Epidemiology Department, National Institute for Infectious Diseases “Lazzaro Spallanzani” I.R.C.C.S., Rome, Italy; 4 Istituto Zooprofilattico Sperimentale delle Venezie, Legnaro, Padova, Italy; Public Health Agency of Canada, Canada

## Abstract

Human cases of infection due to a novel swine-origin variant of influenza A virus subtype H3N2 (H3N2v) have recently been identified in the United States. Pre-existing humoral and cellular immunity has been recognized as one of the key factors in limiting the infection burden of an emerging influenza virus strain, contributing to restrict its circulation and to mitigate clinical presentation. Aim of this study was to assess humoral and cell-mediated cross immune responses to H3N2v in immuno-competent (healthy donors, n = 45) and immuno-compromised hosts (HIV-infected subjects, n = 46) never exposed to H3N2v influenza strain. Humoral response against i) H3N2v (A/H3N2/Ind/08/11), ii) animal vaccine H3N2 strain (A/H3N2/Min/11/10), and iii) pandemic H1N1 virus (A/H1N1/Cal/07/09) was analysed by hemagglutination inhibition assay; cell-mediated response against the same influenza strains was analysed by ELISpot assay. A large proportion of healthy and HIV subjects displayed cross-reacting humoral and cellular immune responses against two H3N2v strains, suggesting the presence of B- and T-cell clones able to recognize epitopes from emerging viral strains in both groups. Specifically, humoral response was lower in HIV subjects than in HD, and a specific age-related pattern of antibody response against different influenza strains was observed both in HD and in HIV. Cellular immune response was similar between HD and HIV groups and no relationship with age was reported. Finally, no correlation between humoral and cellular immune response was observed. Overall, a high prevalence of HD and HIV patients showing cross reactive immunity against two H3N2v strains was observed, with a slightly lower proportion in HIV persons. Other studies focused on HIV subjects at different stages of diseases are needed in order to define how cross immunity can be affected by advanced immunosuppression.

## Introduction

Cases of infection due to a novel swine-origin variant of influenza A virus subtype H3N2 (H3N2v) have recently been identified in the United States. The virus contains genes originating from swine, avian, and human viruses, including the M gene from influenza A(H1N1)pdm09 [1]. Influenza A(H3N2)v viruses are antigenically distinct from seasonal influenza viruses and similar to the proposed swine origin influenza virus (SOIV) vaccine A/Minnesota/11/2010 [1]. Swine to human transmission of A(H3N2)v viruses was rare in 2009-2011 [2]–[5], but increased since July 2012 [6] and sporadic person-to-person transmission was suggested in some cases [7].

Most H3N2v patients experienced mild, self-limited influenza-like illness, and several hospitalized patients presented risk factors for increased influenza complications [8], [9]. The recent increase in human cases of influenza A(H3N2)v virus highlights the need to assess whether human population, unexposed to this variant virus, presents humoral and cell-mediated cross-immunity against A(H3N2)v virus. Indeed, broad immunity established within the host population may have a crucial impact on the burden of infections by an epidemic influenza virus strain, contributing to restrict its circulation and to mitigate clinical presentation. Previous serological studies, focused on antibody cross reactivity against H3N2v, showed an average seroprevalence of 40%, but higher prevalence rates, up to 70%, were found in young adults; unexpectedly, the most susceptible age groups were children and people around 50 years-olds [10]. Seasonal vaccination failed to substantially increase antibody titer against H3N2v in humans [11], and failed to induce protection in ferret animal model [12], [13], suggesting that a specific vaccine may be mandatory if H3N2v epidemic spreads.

The induction of effective T-cell immunity by natural influenza infection as well as by vaccination represents a key step for reducing symptoms severity and preventing complications [14]–[17]. Indeed, a profound impairment of T-cell response was associated with severe or fatal clinical course of pandemic 2009 H1N1 infection, suggesting a strict link between T-cell competence and clinical severity of pandemic influenza infection [18]. Moreover, it was shown that T-cell immunity plays a pivotal role in the context of cross-immunity for its ability to recognize highly conserved internal epitopes [19], thus contributing to expand the repertoire of influenza strains recognition [20].

HIV infection represents a risk factor for greater seasonal influenza-related morbidity and mortality, but the ability to recognize new emerging influenza strains by cross-immunity mechanisms is largely unknown in well-controlled HIV patients [21]–[23]. Recently, it has been shown that HAART-treated HIV patients display both humoral and cell mediated cross-immunity against pandemic H1N1 virus that could be boosted by seasonal vaccination [24]. No data are available on cell-mediated cross-immunity against emerging H3N2v strain both in healthy and in HIV-infected subjects.

Aim of this work was to compare humoral and cell-mediated cross-immunity against two different H3N2 strains (A/H3N2/Min/11/10 and A/H3N2/Ind/08/11) in two samples of presumably unexposed healthy individuals and HIV-infected patients. Moreover, possible association between age and extent of humoral and/or cell-mediated T-cells response was evaluated.

## Materials and Methods

The study was approved by the INMI Ethical Committee (approval number: 81/2012) and all participants gave written informed consent.

### Study population

Healthy donors (HD, n = 45) and HAART-treated HIV patients (HIV+, n = 46) were enrolled at the Spallanzani Institute. The study was approved by the INMI Ethical Committee (approval number: 81/2012) and all participants gave written informed consent. Baseline characteristics of HD and of HIV+ are reported in Table 1. All enrolled HD were negative for HIV, HBV and HCV infections. Inclusion criteria for HIV+ were: CD4>500/mmc and HIV-1 RNA<40 cp/ml. All HIV+ were under effective cART.

**Table 1:** Characterization of enrolled subjects.

	n	Mean Age (years)	Gender (M/F)	Mean CD4/mmc (range)
**HDs**	45	49	16/28	n.t.
**HIV+**	46	51	33/12	717.9 (568–1216)

### Viral strains and antigens

The following influenza A virus strains were used: A/H3N2/Minnesota/11/2010, and A/H3N2/Indiana/08/2011, virtually differing only for the Protein M, and A/H1N1/California/07/2009.

Viral stocks used in HAI were amplified on Madin-Darby Canine Kidney cells genetically modified to over-express α-2,6-linked sialic acid (MDCK-SIAT1, kindly provided by F. Baldanti, Pavia, Italy) in Dulbecco's-modified-Eagle's medium (D-MEM) containing 2 µg/ml of TPCK-treated trypsin (SIGMA) at 35°C in a 5% CO_2_ humidified atmosphere. The MDCK-SIAT1 cell line has been previously described [25].

The haemagglutination titers of the viral stocks were determined using group 0 fresh human blood red cells according to the WHO protocol [26]. The viruses were inactivated by UV light exposure (10 min) and stored at −80°C until use. Complete inactivation of UV-exposed viruses was checked by infecting MDCK-SIAT1 monolayers with undiluted preparations and by back titrating the infectivity after 5 days of incubation.

### Hemagglutination inhibition assay (HAI)

All serum samples were tested by haemagglutination inhibition (HAI) assay to detect A/H3N2/Min/11/10, A/H3N2/Ind/08/11 and A/H1N1/Cal/07/09 HA antibodies respectively. HAI was performed in V-bottom 96-well plates using group 0 fresh human blood red cells [26].

All specimens were tested in serial 2-fold dilutions (from 1∶10 up to 1∶1280). The strain-specific HAI antibody titers for each individual were calculated as the reciprocal (e.g. 80) of the highest serum dilution (e.g. 1∶80) that inhibited haemagglutination. For statistical evaluation, HAI titers below the limit of detection (i.e. 10) were denoted as half of the threshold detection value (i.e. 5); the cut-off HAI titer for protection was set at 40 according to [27], [28].

### Cell mediated immunity

The cell-mediated immune response was assessed by detecting interferon-gamma (IFN-γ) production using an enzyme-linked immunosorbent spot-forming cell assay (ELISpot). Peripheral blood mononuclear cells (PBMCs) were isolated by density gradient centrifugation (Ficoll-Isopaque, Pharmacia Biotech, Piscataway, NJ) and frozen in liquid nitrogen, according to standard procedures, until use. PBMCs were thawed in culture medium (RPMI 1640, 10% FCS, 2mmol/liter L-glutammine) and assessed for vitality by Trypan Blue exclusion, counted, and plated at 3×10^5^ cells/well in ELISpot plates (AID GmbH, Strabberg, Germany). Then, PBMCs were stimulated for 20–24 hours with a multiplicity of infection (MOI) of 0.1 of A/H3N2/Min/11/10, A/H3N2/Ind/08/11 or A/H1N1/Cal/07/09 influenza viruses in the presence of 1 µg/ml αCD28 and αCD49d (IgG1, clones CD28.2 and 9f10, respectively; Becton Dickinson, Mountain View, CA). At the end of incubation, the ELISpot assay was developed according to manufacturer's instructions. Spontaneous (background) cytokine production was assessed by incubating PBMC with αCD28 and αCD49d. Results were expressed as spot forming cells (SFC)/10^6^ PBMCs in stimulated cultures, after having subtracted background. Forty SFC, i.e., average background response + 2SD, was arbitrarily established as the cut-off SFC value for a positive T-cell response, as previously published [24].

### Statistical analysis

The differences between HD and HIV+ subjects were assessed through Kruskal-Wallis equality-of-populations rank test. Association between age and either HAI or T-cell response was assessed by linear regression model. Departure from linear trend was assessed by model based likelihood ratio test (LTR) to assess the model including age as polynomial predictor. In this way the more complex model (i.e. those including age with higher exponent) was preferred over the simple one if LRT p-value was <0.100. Statistical evaluation was performed by STAT 12 statistical package.

## Results

### Humoral cross-immunity against different H3N2v strains in HD and HIV patients

**Figure 1:**
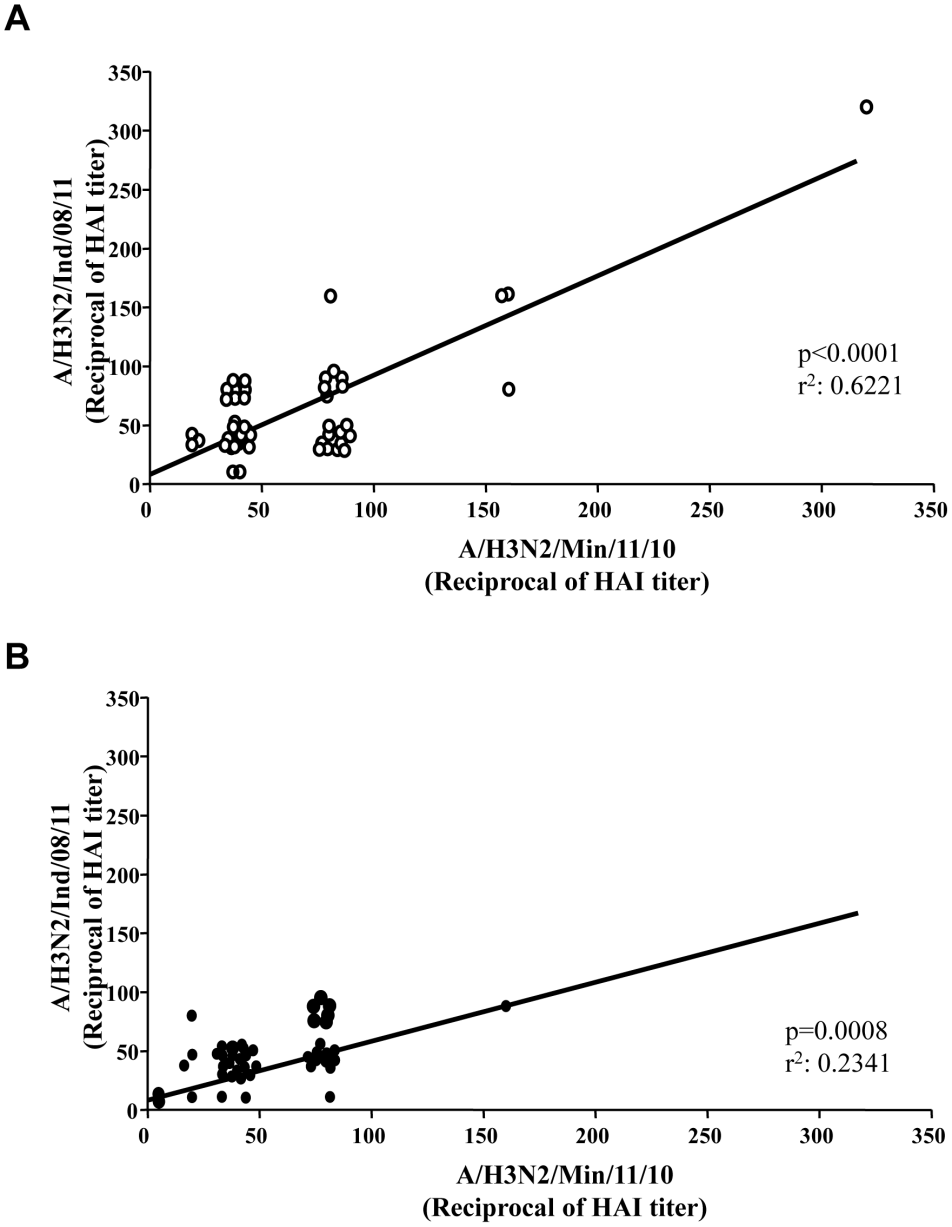
Correlation between humoral immune response to H3N2v strains. (A): The correlation between HAI titers to A/H3N2/Min/11/10 and to A/H3N2/Ind/08/11 is shown in HD. Pearson's r^2^ and significance, as well as the calculated regression line. (B): The correlation between HAI titers to A/H3N2/Min/11/10 and to A/H3N2/Ind/08/11 is shown in HIV+. Pearson's r^2^ and significance, as well as the calculated regression line.

Hemagglutination-inhibition (HAI) antibody titers against two H3N2v strains (A/H3N2/Min/11/10 and A/H3N2/Ind/08/11) were analyzed in 45 presumably unexposed healthy donors (HD) and in 46 presumably unexposed HIV-infected subjects (HIV+), with well controlled HIV viremia and CD4>500/mmc. The characteristics of enrolled individuals are shown in Table 1. In parallel, HAI titers against pandemic A/H1N1/Cal/07/09 were also assessed. In Table 2, the frequency of subjects showing a protective HAI titer (≥1∶40) in the two groups is shown. The frequency of individuals with protective HAI titers was significantly lower in HIV+ as compared to HD for both A/H3N2/Ind/08/11 (HIV+: 21.7% vs. HD: 44.4%, p = 0.043) and A/H1N1/Cal/07/09 (HIV+: 39.1% vs. HD: 71.1%, p = 0.0055). A similar trend was also observed for A/H3N2/Min/11/10 strain, although not reaching a statistical significance, (HIV+: 39.1% vs. HD: 48.8%). When considering the geometric mean titer (GMT), the results confirmed a reduced humoral response in HIV+ group (Table 2). As shown in Figure 1, an high correlation between the HAI titer against A/H3N2/Ind/08/11 and A/H3N2/Min/11/10 was observed both in HD (Panel A, p<0.0001, r^2^ = 0.6221) and in HIV+ (Panel B, p = 0.0008, r^2^ = 0.2341) subjects, in agreement with the expression of an almost identical HA protein by the two viral strains.

**Table 2:** Humoral immune response to different influenza A virus strains in HD and HIV-infected subjects.

	subjects with protective HAI titer ° % (n)			HAI GMT °°		
	A/H3N2/Min/11/10	A/H3N2/Ind/08/11	A/H1N1/Cal/07/09	A/H3N2/Min/11/10	A/H3N2/Ind/08/11	A/H1N1/Cal/07/09
**HD (n = 45)**	48.8% (22)	44.4% (20) *	71.1% (32) **	57.9	57.9 §	76.4 §§
**HIV+ (n = 46)**	39.1% (18)	21.7% (10)	39.1% (18)	46.6	40.6	45.9

**° HAI titer ≥40.**

**°° geometric mean of HAI titer.**

* Chi-square test:

* p = 0.043.

** p = 0.0055.

§ **: Kruskall-Wallis test.**

§ **:** p = 0.014.

§§ : p = 0.0043.

**Figure 2:**
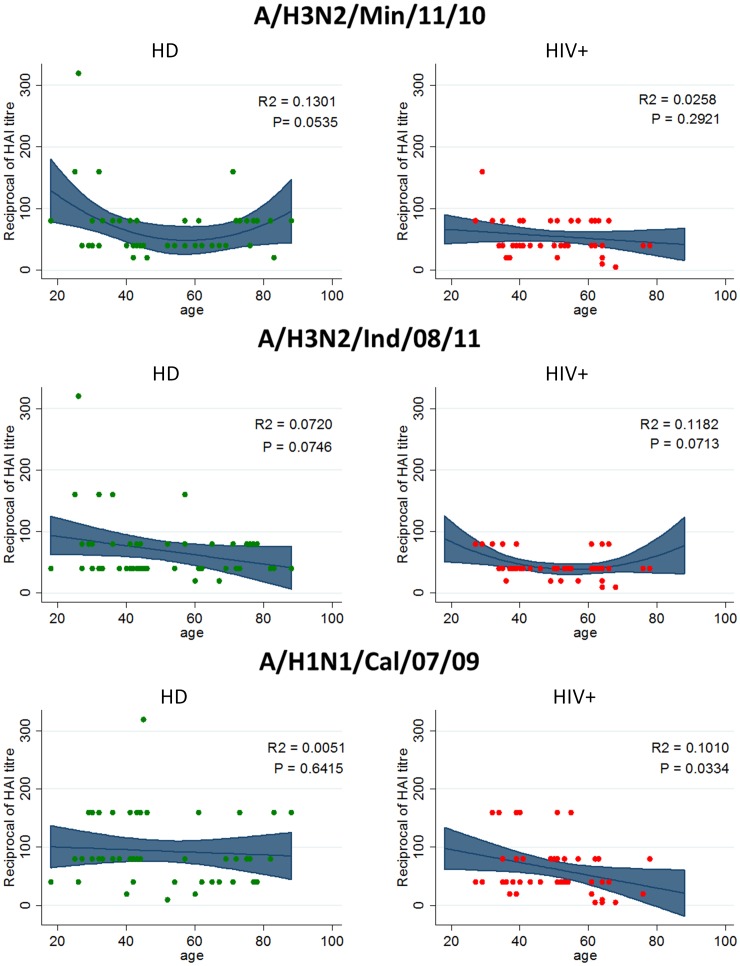
Association between age and humoral immune response to different influenza A virus strains. Age-related relationships of HAI titer against A/H3N2/Min/11/10, A/H3N2/Ind/08/11 and A/H1N1/Cal/07/09 in either HD (left panels) or HIV+ patients (right panels) was assessed by linear regression model. Departure from linear trend was assessed by model based likelihood ratio test (LTR) to assess the model including age as polynomial predictor.

Then possible age-related relationships of antibody titer against all influenza strains were evaluated (Figure 2). A different behaviour of HD and HIV+ groups can be appreciated. Specifically, in HD (Figure 2, Panel A) a weak “U” shaped association between age and HAI titer against A/H3N2/Min/11/10 (r^2^ = 0.1301, p = 0.05) and a potential inverse linear association between age and HAI titer against A/H3N2/Ind/08/11 (r^2^ = 0.072, p = 0.07) were observed. Concerning HIV+ subjects, a weak “U” shaped association between age and HAI titer against A/H3N2/Ind/08/11 (r^2^ = 0.1182, p = 0.07), and a significant inverse linear association between age and HAI titer against A/H1N1/Cal/07/09 (r^2^ = 0.1010, p = 0.03) were shown. On the contrary, no association of age with antibody titer against A/H3N2/Min/11/10 was found in HIV+. The different age-association found for A/H3N2/Ind/08/11 and A/H3N2/Min/11/10, both in HD and in HIV, could be explained by the slight sequence differences (two residues) between HA of the two viruses.

### Cell-mediated cross-immunity against two H3N2v strains in HD and HIV patients

The analysis of cell-mediated cross immunity was performed by ELISpot assay. As shown in Table 3, a large proportion of both HD and HIV+ displayed a significant T-cell response (>50SFCs/10^6^PBMC) to the A/H3N2/Ind/08/11 strain (53.3% and 34.7% respectively) and to the A/H3N2/Min/11/10 (53.3% and 37% respectively). Nevertheless, the proportion of responding individuals was weakly decreased (albeit not reaching statistically significance) in HIV+ as compared to HD, suggesting a similar reduction of humoral and cell-mediated immune response during HIV infection (Table 3). In line with these results, but with much stronger evidence, a decreased frequency of individuals responding to pandemic A/H1N1/Cal/07/09 strain was observed in HIV+ vs HD (HIV+: 37.0% vs. HD: 68.8%, p = 0.0023, Table 3).

**Table 3:** Frequency of HD and HIV+ presenting a T-cell response to different influenza A virus strains.

	A/H3N2/Min/11/10 % (n)	A/H3N2/Ind/08/11 % (n)	A/H1N1/Cal/07/09 % (n)
**HD (n = 45)**	53.3% (24)	53.3% (24)	68.8% (31) **
**HIV+ (n = 46)**	37.0% (17)	34.7% (16)	37.0% (17)

* chi-square test:

**p = 0.0023.

**Figure 3:**
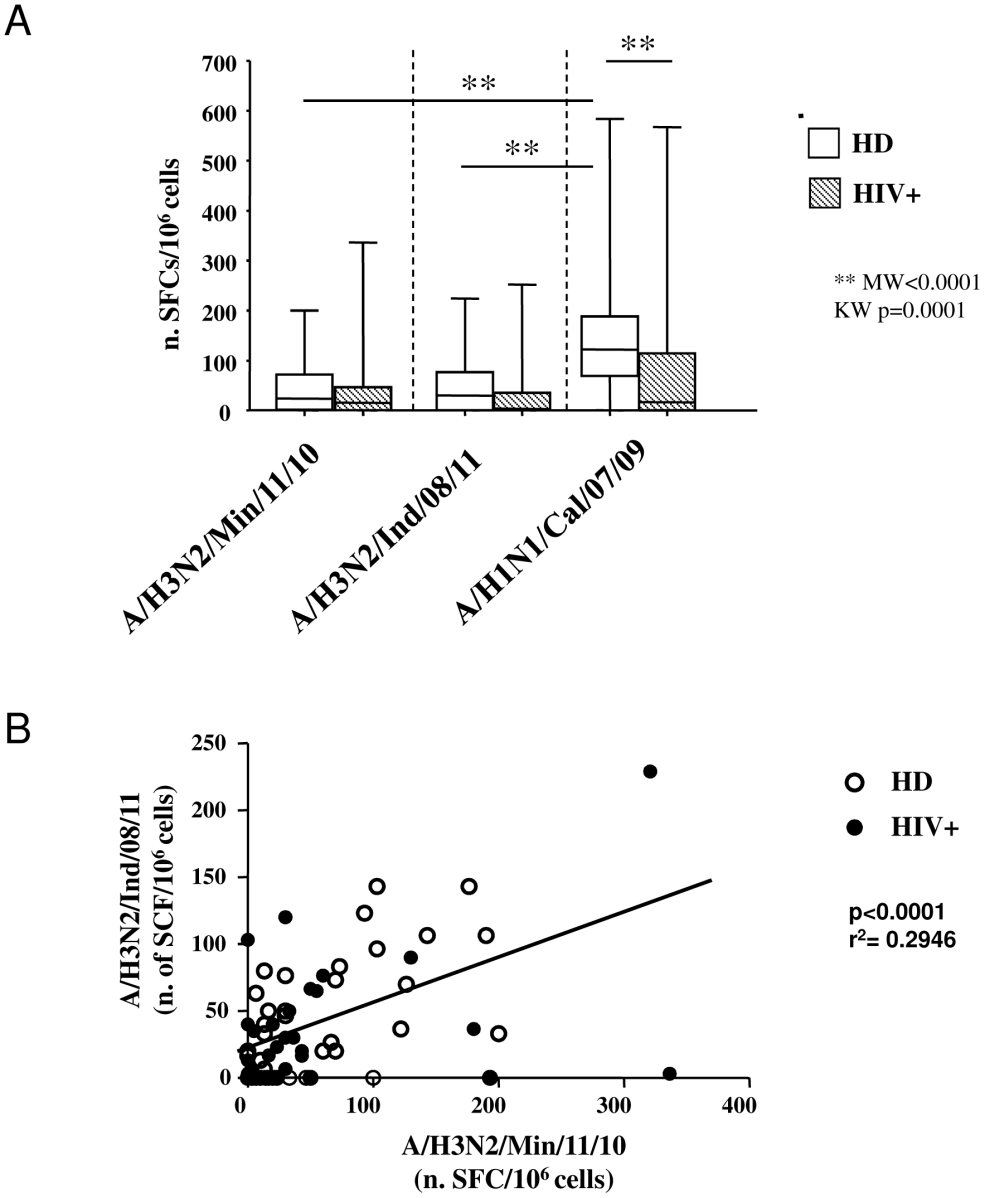
Cellular immune response to different influenza A virus strains. (A): The frequency of T-cell response specific for A/H3N2/Min/11/10, A/H3N2/Ind/08/11 and for A/H1N1/Cal/07/09 was evaluated by ELISpot assay in HD (n = 45, white bars) and HIV+ patients (n = 46 grey bars). Results are indicated as SCFs/10^6^ peripheral mononuclear cells (PBMCs). Statistical analysis was performed using non parametric Mann-Whitney assay. ** p<0.0001. (B): The correlation between T-cell response against A/H3N2/Min/11/10 and A/H3N2/Ind/08/11 is shown, with Pearson's r^2^ and significance, as well as the calculated regression line. HD and HIV+ are reported as white and black circles, respectively.

The extent of cross reactive T-cell immunity against both H3N2v strains was similar in HIV+ and HD (Figure 3A); in contrast, a strongly reduced T-cell response to pandemic A/H1N1/Cal/07/09 strain was shown in HIV+ (HIV: median 16.6 [IQR: 0.00–105.0] SFC/10^6^ cells vs. HD: median 121.7 [IQR: 61.6–188.3] SFC/10^6^ cells, p = 0.0001).

However, as shown in figure 3A, the extent of T-cell response against the three influenza strains was similar in HIV+ subjects, while higher response against A/H1N1/Cal/07/09 was observed in HD (A/H1N1/Cal/07/09: median 121.7 [IQR: 61.6– 188.3] SFC/10^6^ cells, A/H3N2/Ind/08/11: median 20.0 [IQR: 0.0–71.6] SFC/10^6^ cells, A/H3N2/Min/11/10: median 21.6 [0.0–71.6] SFC/10^6^ cells, p<0.0001 for both comparisons).

As shown in figure 3B, a high correlation (r^2^ = 0.2946, p<0.0001) was observed between T-cell response against the two H3N2v strains, differing only for the M protein, suggesting a minor role of this protein in eliciting cellular cross-immunity. Surprisingly, and in line with previous observation [20], no correlation was observed between humoral and cell-mediated immune response for all viral strains (A/H3N2/Min/11/10: r^2^ = 0.0006, p = 0.8197; A/H3N2/Ind/08/11: r^2^ = 0.003961, p = 0.5579; A/H1N1/Cal/07/09: r^2^ = 0.001691, p = 0.7053).

**Figure 4:**
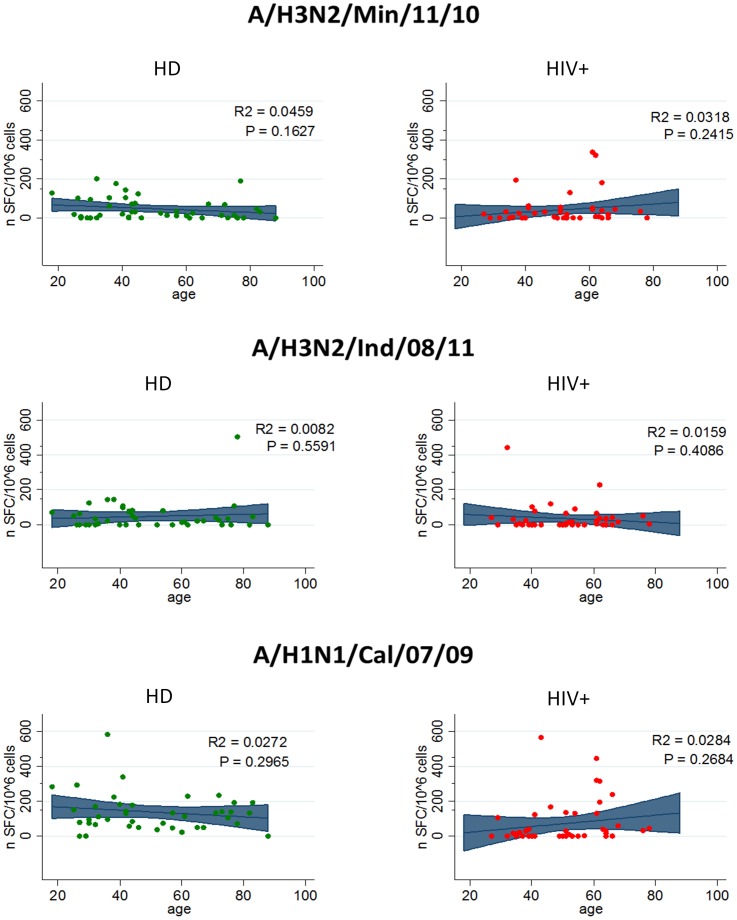
Association between age and cellular immune response to different influenza A virus strains. The potential and the shape of association between age and T-cell response against A/H3N2/Min/11/10, A/H3N2/Ind/08/11 and A/H1N1/Cal/07/09 is shown for HD (left panels) and HIV+ (right panels) was analyzed by linear regression model. Departure from linear trend was assessed by model based likelihood ratio test (LTR) to assess the model including age as polynomial predictor.

Finally, we wondered whether T-cell response may be dependent of age (Figure 4). Differently from humoral response, no statistically significant association was observed, and the age-related distributions of T cell response against the two viral strains were similar (Figure 4).

## Discussion

The emergence of influenza viruses from the animal reservoir is a permanent challenge for public health for their potential introduction into humans and sustained circulation among populations of immunologically naïve, susceptible hosts. Indeed, a pre-existing immunity through both humoral and cell-mediated arms represents an important factor counteracting the pandemic potential of an emerging influenza virus strain.

In this study, a large proportion of healthy and HIV+ subjects displayed cross-reacting humoral and cellular immune response against two H3N2v strains. Specifically, in agreement with published data [10], [29], 44.4% of HD presented a protective HAI titer against A/H3N2/Ind/08/11. Interestingly, we showed for the first time a significantly reduced seroprevalence against A/H3N2/Ind/08/11 and A/H1N1/Cal/07/09 in HIV+; similarly, a decrease was also observed for seroprevalence against A/H3N2/Min/11/10 strain, although not reaching statistical significance. It is possible that small sample size and relatively high variability could account for the lack of significance in the latter case. Moreover, in agreement with the expression of an almost identical HA protein by A/H3N2/Ind/08/11 and A/H3N2/Min/11/10, a high correlation between the HAI responses to both H3N2v strains was observed in HD and in HIV subjects. Phylogenetic analysis indicated that the HA of H3N2v strains descended from H3N2 viruses that were circulating worldwide in humans in the mid-1990s [30]. Thus, the high seroprevalence observed against H3N2v could be explained by previous exposure to this virus and/or to an involvement of cross-immunity mechanisms. Unfortunately, we were unable to investigate the vaccination history of enrolled HD and HIV+, and this may represent a weak point of our study. Nevertheless, several studies reported that seasonal vaccination failed to substantially increase cross-reactive antibodies against H3N2v [11]–[13], partially relieving the weakness of our study concerning this point.

The age-dependent susceptibility to influenza infection may strongly influence epidemic dynamics. In this context, an age dependent prevalence of antibodies cross-reactive to H3N2v was reported [10], [31], showing that the most susceptible groups were children (<10 years) and people around 50 years. To this respect, our age-dependence findings on H3N2v strains are in line with these results, with U shaped curves well supported for A/H3N2/Ind/08/11 in HIV+ and for A/H3N2/Min/11/10 in HD (Figure 2). It is possible small numbers and lack of younger subjects in our study groups may account for the lack of statistical support to age-dependent response in the other instances.

The protection against influenza virus infections was currently evaluated by measuring the antibody response. Nevertheless, in the last years, a main role of cellular immune response was proposed as able to mediate the clearance of circulating and antigenically novel influenza virus infection through strain specific and cross-specific immune mechanisms. In this study, a large proportion of HD and HIV+ presented a T-cell response against both H3N2v strains, novel for human population, and the extent T-cell cross-response to novel H3N2v stains was similar between the two groups, suggesting the existence of cross-reactive T-cell clones both in healthy and HIV+ subjects. Cellular cross-immunity against various influenza virus strains was reported in animal models [32]–[34] and in humans [20], [33], [35]–[37], and it is considered a main mediator of both early clearance of influenza virus and mitigation of clinical severity [14]–[18], [38]. As a matter of fact, while pre-existing T-cell immunity may have contributed to mitigating the impact of the 2009 H1N1 pandemic virus strain [39], a profound impairment of T-cell immunocompetence was associated to a severe/fatal clinical course of pandemic influenza infection [18]. In this study, the HIV+ patients presented suppressed HIV viremia and CD4 number >500/mmc. Nevertheless, T-cell response against A/H1N1/Cal/07/09 in HIV+ was significantly lower than in HD. One possible explanation for this finding may be that even in HIV+ subjects without evident signs of immune compromission (i.e. CD4 >500/mmc) subtle alteration of immune competence may exist, due to the loss of specific T cell clones, as reported in the literature [40]. So, it is not unexpected that T-cell reactivity against a defined influenza viral strain may be significantly reduced as compared to HD. A similar analysis in an HIV+ population with a more advanced disease could be interesting in order to define the role of more pronounced immunosuppression in modulating cross-reactive immunity.

One of the main features of cell-mediated T-cell response is its ability to recognize highly conserved internal epitopes [19], resulting in a broad protection against different strains. On this issue is based the development a universal influenza vaccine targeting conserved internal proteins such as M and NP, able to elicit a broad and long lasting immunity. In order to investigate the contribution of M protein in the cross immunity against H3N2v strains, we performed a comparison between T-cell response against A/H3N2/Ind/08/11 and A/H3N2/Min/11/10, differing only for M protein. A highly significant correlation was found, suggesting a minor role of M protein in inducing cross immunity against H3N2v strains.

To our knowledge this is the first study showing results on humoral and cell-mediated cross-immunity against H3N2v influenza strains in HIV-infected persons. Overall, the main results show high prevalence of HD and HIV+ patients showing humoral and cell-mediated cross immunity against two H3N2v strains, with a slightly lower proportion in HIV+ persons. Moreover, the age seems to represent a factor driving humoral response both in HD and in HIV+ persons but not cell-mediated response. Other studies focused on HIV+ persons at different stages of diseases are needed in order to define how cross immunity can be affected by advanced immunosuppression.
